# Validity of the Self-Expression and Emotion Regulation in Art Therapy Scale (SERATS)

**DOI:** 10.1371/journal.pone.0248315

**Published:** 2021-03-10

**Authors:** Suzanne Haeyen, Eric Noorthoorn

**Affiliations:** 1 GGNet, Centre for Mental Health, Scelta, Expert Centre for Personality Disorders Apeldoorn, Warnsveld, The Netherlands; 2 Research Centre for Arts Therapies and Psychomotricity for Personality Disorders & Master of Arts Therapies, HAN University of Applied Sciences, Nijmegen, The Netherlands; 3 KenVaK, Research Centre for the Arts Therapies, Heerlen, The Netherlands; 4 Radboud University, Nijmegen, The Netherlands; University of Twente, NETHERLANDS

## Abstract

**Background:**

The Self-Expression Emotion Regulation in Art Therapy Scale (SERATS) was developed as art therapy lacked outcome measures that could be used to monitor the specific effects of art therapy. Although the SERATS showed good psychometric properties in earlier studies, it lacked convergent validity and thus construct validity.

**Method:**

To test the convergent validity of the SERATS correlation was examined with the EES (Emotional Expressivity Scale), Emotion Regulation Strategies for Artistic Creative Activities Scale (ERS-ACA) and Healthy-Unhealthy Music Scale (HUMS). Patients diagnosed with a Personality Disorder, and thus having self-regulation and emotion regulation problems (*n =* 179) and a healthy student population (*n =* 53) completed the questionnaires (*N =* 232).

**Results:**

The SERATS showed a high reliability and convergent validity in relation to the ERS-ACA *approach strategies* and *self-development strategies* in both patients and students and the HUMS *healthy scale*, in patients. Hence, what the SERATS measures is highly associated with emotion regulation strategies like acceptance, reappraisal, discharge and problem solving and with improving a sense of self including self-identity, increased self-esteem and improved agency as well as the healthy side of art making. Respondents rated the SERATS as relatively easy to complete compared to the other questionnaires.

**Conclusion:**

The SERATS is a valid, useful and user-friendly tool for monitoring the effect of art therapy that is indicative of making art in a healthy way that serves positive emotion regulation and self-development.

## Introduction

Art therapy, a treatment based on the experience and the use of art media in a methodically targeted manner, is used quite frequently in mental healthcare [1]. Patients as well as professionals consider it important and meaningful [e.g. 2–5] and a limited number of effect studies show the effectiveness of art therapy [4–7]. Art therapy offers a space for exploration and experiencing emotions and/or (inner) conflicts. Feelings or themes that are difficult to process or handle can be explored without being directly expressed in words, as described in many handbooks on art therapy [e.g. 3, 8–10]. The working with art materials is experiential and often offers a trigger for activating emotions [10–15]. Although these clinical experiences and the few performed effect studies suggest art therapy is an effective intervention, it lacks outcome measures to monitor and evaluate the specific effects related to it [5, 16–18]. Art therapy is mostly integrated in such programmes. A specific scale for measuring art therapy in multidisciplinary programs is needed because such scale isolates the contribution of art therapy as a part of a broader programme. If results are more isolated these can provide situation-specific feedback. This makes it possible to stimulate the quality of art therapy by intervening in certain aspects and/or by using the results to discuss the therapy process with the service-users for evaluation and improvement. More general scales do not fit well art therapy and the involved processes. A specific scale improves insight in the contribution of art therapy to the treatment process as such. Our aim is to develop an instrument to measure perceived effects of art therapy among patients with emotional and self-regulation problems.

In literature there are some related but not very suitable outcome measures to monitor and evaluate art therapy. The few available diagnostic scales, such as the Diagnostic Drawing Series [19] or the Art-Based Intervention self-report questionnaire on the art-making experience [16], are assessment or observation tools and not specifically suited to measure the effects of art therapy treatment [20]. Other scales do not fit well the *therapy* aspect of art therapy or do not fit process of art-making. For example, the recently developed instrument focused on artistic creative activities called `Emotion Regulation Strategies for Artistic Creative Activities Scale’ (ERS-ACA) measures ERSs when engaging in artistic creative activities [21]. That study focused on “design or crafts” activities including even gardening, baking or cooking as ‘artistic’ activities, rather than on (art) therapy and it involved a general healthy sample rather than a clinical sample. A scale about emotion regulation but not about the process of art-making is the Emotion Regulation Questionnaire (ERQ) [21]. This is a 10-item scale on a 7-point Likert scale that focuses on ERSs in daily life (rather than related to a specific activity). The scale has two subscales: ‘cognitive reappraisal’ and ‘expressive suppression’. The ‘cognitive reappraisal’ subscale includes distraction and calm focus on the problem. It does not include broader cognitive strategies such as problem solving or acceptance [22]. The scale focuses on these specific strategies. This makes the scale narrow in scope. Even more important, the items do not fit the specific character of art therapy. Finally, the Cognitive Emotion Regulation Questionnaire (CERQ) focuses on coping following negative situations or events and includes subscales on emotional regulation strategies such as rumination and reappraisal, but also on more specific subscales such as self-blame, other-blame and catastrophizing [23]. Although interesting, it also does not fit the specific context of art therapy. Consequently, there is a gap for a new validated scale for measuring the results of art therapy.

As no instruments were available to measure the specific effects of art therapy, we used developed effect categories from previous research [2] as input for the development of an assessment tool for examining the contribution of art therapy in the multidisciplinary treatment of personality disorders. These effect categories were developed on the patients’ experiences with art therapy, expressed during individual and focus group interviews. The development of the Self-Expression and Emotion Regulation in Art Therapy Scale (SERATS) was reported [24].

Emotion-regulation problems and emotional vulnerability are key issues for personality disorders [5, 25, 26]. Emotion regulation refers to the processes of how people influence experienced emotions: when we have emotions, how we experience them and how we express them [27]. The way people express emotions plays an important role in social interactions [28, 29]. Personality disorder patients often suffer from weakened autonomy and an unstable, diffuse or negative self-image. They often encounter difficulties with self-determinism in the sense of impulsiveness, how to handle distance and closeness, and achieving personal goals. They also often have difficulties with interpersonal relationships, with being able to feel connected and with intimacy. This often involves destructive and/or self-destructive behavior, suicidal thoughts, negative or distorted cognitions, and affective deregulation. They tend to be overwhelmed by emotions, have a high level of tension, get agitated quickly, and find it difficult to calm themselves down [5].

In the art process, the personality disorder patient is responsible for the creation of the art product, and this asks for self-management or self-direction [5]. Art therapy may help patients to recognize difficult emotions, to integrate conflicting thoughts, feelings or behaviors and to find a more constructive way of dealing with them [30]. In art therapy, emotional states can be triggered by what happens during the art process and the interaction with the art materials [7]. The most improved treatment goals of art therapy, reported by patients diagnosed with a personality disorder in a quantitative survey (*N =* 528) were: expression of emotions, improved (more stable/positive) self-image, making own choices/autonomy and, recognition of, insight in and changing of personal patterns of feelings, behaviors and thoughts [30]. In art therapy, the inner self becomes visible, tangible and concrete through the work produced. It may be a conscious or unconscious process, and the meaning is always determined in a dialogue with the client [2, 5, 8]. The artwork mirrors aspects of the patient, both by the content of the image as by its formal image characteristics as many experts agree. Art therapy seems to help the handling of emotions in allowing a slow, manageable pace to the organisation of thoughts and feelings through art making and a stepwise process of converting these into words and then communication with others in a group discussion. The art therapy process supports explicit and implicit mentalisation [e.g. 31, 32]. The SERATS was developed based on previous studies [2, 5, 24, 35] to capture art therapy specific effects, related to emotion regulation and self-expression, such as resolving inner conflicts [33], awareness of the here and now [31], and achieving personal insight [34].

Patients with a personality disorder cluster B are often overwhelmed and entangled in their intense emotions. In response, they make every effort not to feel, often by exhibiting destructive behavior. Cluster C patients are often afraid to rely on their feelings and start avoiding them. They visibly or invisibly withdraw from contact (with themselves and with the other). What is consistent in terms of emotion regulation is that these patients often do not know what they are feeling anymore, and the therapeutic process is aimed at reconnecting with underlying feelings first. Only when this emotional awareness of what they are experiencing here and now is there (see Table 1, SERATS item 1), someone can increasingly notice that they can express feelings in the artwork (item 2). On the basis of this, they may experience that they are beginning to discover and understand themselves and the situations they are dealing with better (item 3,4,5) and that they can discharge themselves through art making (item 6). An art product can help to maintain an experience or feeling (item 7) so that more continuity can be experienced. Final results of this process can become apparent in the application of new behaviors outside of art therapy (item 8) and that they acquire more self-insight (item 9). This process in art therapy is about becoming more aware, being able to give direction and thereby processing difficulties. In art therapy, this is initially achieved through an experiential route, by bottom-up emotion regulation. This experience leads to behavior change and an improved self-understanding.

**Table 1:** The nine items of the SERATS (for a user version see the S1 Appendix).

Self-Expression and Emotion Regulation Scale
1. I get in touch with my feelings through the process of making art
2. I am able to depict my feelings in art therapy
3. Through the process of making art, I am able to discover what is at play within me
4. I am able to express my feelings through the process of making art
5. I am able to make things fall into place in the art
6. Making art is a kind of outlet for me
7. A piece of art I have created can help me hold on to a particular feeling
8. I apply the new behavior that I have been experimenting with in art therapy outside of the therapy setting
9. I gain greater insight into my psyche through art therapy

The context specific SERATS is constructed in a series of analyses [24], in which the psychometric properties of this scale were tested, using two independent patient samples, respectively (*n =* 335, *n =* 34). Context specific because all items describe what happens to a patient in the context of art therapy. Patients who participated had been diagnosed with personality disorders of clusters B or C. The exploratory and confirmatory factor analyses resulted in a unidimensional 9-item scale with item factor loading above .7. The content of the final items is focussed on experiencing, becoming aware and expressing feelings, regulating emotions/feelings (letting out, making fall into place or holding on to) by applying new behavior and gaining insight, all in relation to the art therapy experience. Because the focus of the scale is self-expression and emotion regulation, we named the scale the SERATS.

The scale showed a high internal reliability with a Cronbach’s alpha of .94 and high test-retest reliability of *r =* .96. In other studies, we examined if the SERATS-scores also changed, in a pretest-posttest design and in a randomised clinical trial (RCT) with an isolated art therapy intervention [5, 35] to investigate if the change in the SERATS could be attributed uniquely to art therapy This meant that patients only received art therapy and no other interventions. We used GLM repeated measures analyses and computed change of the effect size in Cohen’s *d* (Δ *d*) between the effect size at the post-test and the effect size at the pre-test. The effect of time was significant for SERATS, F (1, 31) = 111.55, *p =* .00. In addition, the effect size was very large, *d =* 2.03. Therefore, we concluded that the SERATS was sensitive to change in art therapy, which was in line with the earlier results [35].

We also explored the validity of the SERATS in two studies [24, 35] by examining the association between de SERATS and a range of measures. We examined the correlations between the SERATS and the AAQ-II (psychological flexibility, i.e., the Acceptance and Action Questionnaire-II [36], MAAS (Mindfulness Attention Awareness Scale) [37], the OQ45 (general mental functioning, i.e., Outcome Questionnaire 45) [38], the SMI (presence of different maladaptive and adaptive mental modes) [39], and the MHC-SF (social-, emotional-, and psychological well-being, i.e., Mental Health Continuum—short form) [40]. The results showed that the SERATS did not correlate enough with these outcomes (*r* < .4). These results indicated discriminant validity of the SERATS but lacked convergent validity. Convergent and discriminant validity are both considered subcategories or subtypes of construct validity. The important thing to recognize is that they work together. If there is evidence for both convergent and discriminant validity, then by definition is demonstrated that there is evidence for construct validity. The previous studies focusing on the SERATS examined the psychometric qualities of this new questionnaire. Only if the SERATS emerged as reliable and valid we wanted to encourage the use of the SERATS in other studies.

Although the SERATS showed good psychometric properties, it lacked construct validity. The measures we used in the earlier studies were chosen for testing the effects of art therapy. They were not specific for a validity study. It could be theoretically possible that the lack of convergent validity was related to a wrong choice of measures. We used general questionnaires about emotional functioning in previous studies [24, 35]. One of these with a subscale `emotional functioning’ (OQ45; Outcome Questionnaire 45) and another about the presence of emotional modes, which can be well understood as emotion regulation strategies (SMI; Schema Mode Inquiry). However, correlations between these scales and the SERATS were too small to determine convergent validity. To validate the SERATS we needed questionnaires close to the content of the context specific SERATS.

This asked for further investigation and the use of more related outcome measures [5, 35]. Convergent validity is important to in defining what the SERATS measures exactly. In this study we examine the convergent validity of the SERATS using specific outcome measures on self-expression and regulation of emotions.

The primary aim of the current study is to examine the *validity* of the Self-expression and Emotion Regulation in Art therapy scale (the SERATS). When the SERATS is validated we will discuss practical use and advantages. A well validated scale focused on self-expression and emotion regulation designed for art therapy would allow us to understand how art therapy is received and thereby support the design of interventions to enhance the regulation of emotions (in the short-term), mood (in the mid-term) and mental health (in the longer-term). This is an important goal given that art therapy showed in a number of studies to be particularly effective in regulating emotions, mood and mental health [5, 41–43]. There is increasing use of them as adjunct therapies within mental health and as recommended daily activities to support wellbeing [44–46].

## Materials and methods

The objective of this explorative study was to test the convergent validity of the SERATS (its correspondence with measures of a similar construct). Therefore, three other questionnaires focused on emotion regulation and self-expression were selected.

### Participants and procedure

Adult patients (18–65 years) admitted to a specialized treatment program of personality disorders. All patients were diagnosed with one or more personality disorders as a primary diagnosis (*n =* 179). The age of these participants varied from 18 to 67 years with a mean of 37.66 years (SD = 12.8 years); 106 participants were female (70.9%), 43 were male (17%). Next to the diagnoses of PD cluster B (80%), C (63%) and PD NOS (not otherwise specified) (70%) there were comorbid diagnosis of mood (81.5%) and anxiety disorders (91.3%). Many patients reported to be diagnosed with more than one PD/comorbid disorders.

A healthy population not being treated for mental problems also participated. A group of students (18 years and older) completed the questionnaires (*n =* 53). These were students of a bachelor’s program in art therapy and were therefore active in the visual arts. The age of these participants varied from 18 to 59 years with a mean of 24.20 years (SD = 7.94 years); 51 participants were female (96.2%), 2 were male (3.8%). This yielded complete responses from 232 participants, which were used for analyses of convergent validity and correlation with related constructs.

Only patients receiving art therapy treatment as a part of the specialized treatment could complete the SERATS and were included in this study. The one-off collection of the four questionnaires (duration max. 20 minutes) was administered at one point in time immediately after an art therapy session. This was expected to have no risks for the participants. Participation in this study was completely voluntarily, and participants were allowed to stop participating without any clarification. The questionnaires were handed out to patients and students by therapists and teachers of their institutions. Each participant signed an informed consent letter.

All procedures performed in the current study were in accordance with the 1964 Helsinki declaration and its later amendments; and with comparable ethical standards. No patient data was recorded. Data were analysed based on fully anonymized data that allowed none of the cases to be traced to an individual. No ethical approval was required in accordance with Dutch law, as no intervention was carried out. Institutional ethical and legal check according to Dutch law and procedures was performed by the hospitals data protection officer, a Master of Law.

### Measures

The questionnaires administered alongside the SERATS are the EES (Emotional Expressivity Scale), the Emotion Regulation Strategies for Artistic Creative Activities Scale (ERS-ACA) and the Healthy-Unhealthy Music Scale (HUMS).

The **Emotional Expressivity Scale** (EES) is a measure of individual differences in the degree to which people outwardly express their emotions rates the emotions someone displays outwardly across a range of severity [47]. The EES does not indicate specific positive or negative emotions but describes general expressiveness of emotions [48]. The EES is a self- report measure which contains 17 items. The scale includes example items such as “I display my emotions to other people” and “People can read my emotions”. The participants respond on a 6-point Likert scale that varies from 1 = never true to 6 = always true. To calculate the scores, the average is taken, with a higher score meaning greater emotional expressiveness. The EES shows convergent and discriminant validity that have been established through self-reporting, other reporting and through observation methods [49].

The **Emotion Regulation Strategies for Artistic Creative Activities Scale** (ERS-ACA) measures types of emotional regulation strategies (ERSs) used when engaging in artistic creative activities [21]. This is an 18-item scale with an overall ‘general’ factor of emotion regulation strategies alongside three subscales: a 7-item factor comprising ‘avoidance strategies’ (such as distraction, suppression and detachment), a 6-item factor comprising ‘approach strategies’ (such as acceptance, reappraisal and problem solving), and a 5-item factor comprising ‘self-development strategies’ (such as enhanced self-identify, improved self-esteem and increased agency). Example items for subscale 1. `Avoidance strategies’ are: “it directs my attention so I forget unwanted thoughts and feelings”, and “it helps me to disengage from things that are bothering me”. For subscale 2. `Approach strategies’: “it helps me to come to terms with my own emotions” and “I can contemplate what is going on in my life with a clear mind”. For subscale 3. `Self-development strategies’: “it reaffirms my identity” and “it gives me a sense of purpose”. The factor analysis (*n =* 740, *n =* 47924) showed strong internal reliability (Cronbach’s alpha: General Factor = 0.93, Factor 1 = 0.9, Factor 2 = 0.88, Factor 3 = 0.88). The scale showed convergent and divergent validity, construct validity, consistency of internal reliability and test-retest reliability. The scale possesses high internal reliability and consistency of reliability, good convergent and divergent validity, clear construct validity, and strong test-retest reliability (*n =* 165) [21].

The **Healthy-Unhealthy (music) Scale** (HUMS) consists of 13 items, which measures the healthy and unhealthy dimensions of musical engagement [50]. The scale is divided into the Healthy and Unhealthy subscale. The reliability and validity tests provided favorable and confirmative results. Cronbach’s alpha coefficients were .78 for Healthy and .83 for Unhealthy. The concurrent validity of the HUMS was confirmed through correlations to wellbeing, happiness and school satisfaction on one hand and depression, rumination, and stress on the other. For the Health-Unhealthy Music scale the word ‘music’ was modified to ‘art making’. Example items for Unhealthy subscale are: `When I am making art, I get stuck in bad memories’ or `Art making gives me an excuse not to face up to the real world ‘ and examples for the Heathy subscale is: ‘Art making gives me the energy to get going’ or `I feel happier after art making’.

The **Self-Expression and Emotion Regulation in Art Therapy Scale** (SERATS; [24]) is a one-factor instrument that measures self-expression and emotion regulation in art therapy. The instrument consists of nine items (e.g. “I am able to depict my feelings in art therapy.” and “Making art is kind of an outlet for me.”), that are scored on a 5-point Likert scale running from never true (1) to almost always true (5). The reliability is excellent with a Cronbach’s alpha of .94 and high test-retest reliability of r = .96. The SERATS is sensitive to change. The scale showed discriminant validity but convergent validity is not yet established [24].

#### Translation process

We used versions of the instruments in the Dutch language which were available for the EES and the SERATS. The instrument translation process of the HUMS and the ERS-ACA included back-translation [51]. This means that the instrument used was translated from the source language into the target language by a translator. Then the target language version was translated back into the source language by other translators. Then, the two source language versions were compared. Semantic equivalence between source language version and target language version can be verified. Direct comparison of two source language versions provides additional evidence of quality.

#### Extra question about the experienced difficulty

An additional question was added to each questionnaire regarding how the questionnaire was experienced to be completed, with a 10-point scale answer option ("very easy" to "very difficult").

### Analysis

All analyses were performed with SPSS 22 [52]. Convergent validity was evaluated by inspection of the Pearson correlations between the SERATS and the other measures. The sample was determined on the basis of the number of dimensions, whereby there must be at least 20 subjects per dimension [53]. Because in all scales there are 8 scales together, we ended up with a minimum of 160 participants. We also wanted to compare these results with a healthy group. For this, the minimum number was set at 50 [53]. The target correlation we wanted to find was >. 4 [54]. Pearson’s r correlation coefficients were used to explore the relationship between the SERATS and the other related scales focused on emotion regulation and self-expression. Cronbach’s alphas were computed for the scale to confirm the stability of internal consistency of the scale. The answers on the additional question about the experienced difficulty was analyzed with frequencies.

## Results

The data showed a normal distribution (skewness/kurtosis) on the item-level as well as the level of the (sub)scales.

### Reliability analysis

We first evaluated the internal consistency (with Cronbach’s alpha) of the (sub)scales in the complete dataset with all respondents (patients and students) and after that for the dataset split in the subgroups. The SERATS showed a high reliability with a Cronbach’s Alpha of .90. The ERS-ACA also showed high reliability (.87 to .91). The EES and the HUMS showed far lower reliabilities with respectively .49 and .73. The results are shown in Table 2.

**Table 2:** Reliability of the SERATS, the EES, ERS-ACA and the HUMS.

	Cronbach’s Alpha (*n =* 232)
SERATS	.90
EES	.49
ERS-ACA total	.91
subscale Avoidance	.87
subscale Approach	.88
subscale Self-development	.91
HUMS subscale Unhealthy	.74
subscale Healthy	.73

### Construct validity

We did not have ante hoc hypotheses about how strongly the SERATS should correlate with the outcomes, because this study is explorative. The target correlation we wanted to find was >. 4 [54]. The correlations between the SERATS on the one hand and the EES, ERS-ACA, and HUMS on the other hand were the highest for the ERS-ACA *approach strategies* with r = .69 in the patient sample and .81 in the student sample. The ERS-ACA *self-development strategies* showed less high correlations with the SERATS but still >.5 and significant at the 0.01 level in both samples. It is striking that the student sample shows a high correlation between the SERATS and the ERS-ACA *avoidance strategies* (.57 **) in contrast to the patient sample. The student sample also shows relatively a high but negative correlation between SERATS and emotional expressivity (EES). The patient sample shows a high and significant correlation between the SERATS and the HUMS *healthy scale* (see Table 3).

**Table 3:** Correlations of the SERATS with the EES, ERS-ACA, and HUMS.

Participants			EES Totaalscore	HUMS unhealthy	HUMS healthy	ERS-ACA avoidance strategies	ERS-ACA approach strategies	ERS-ACA self-development strategies
Patients (n = 179)	SERATS totaalscore	Pearson Correlation	-.08	.22**	.57**	.10	.69**	.53**
		Sig. (2-tailed)	.36	.01	.00	.22	.00	.00
Students (n = 53)	SERATS totaalscore	Pearson Correlation	-.48**	-.23	.26	.57**	.81**	.62**
		Sig. (2-tailed)	.00	.12	.09	.00	.00	.00
All (n = 232)	SERATS totaalscore	Pearson Correlation	.29**	.11	.57**	.20**	.72**	.58**
		Sig. (2-tailed)	.00	.13	.00	.00	.00	.00

* Correlation is significant at the 0.05 level (2-tailed).

** Correlation is significant at the 0.01 level (2-tailed).

### Experienced difficulty with the questionnaires

The answers on the additional question about the experienced difficulty were analyzed with frequencies. Table 4 shows that most people experienced the SERATS as relatively `easy’ to complete (mean 4.58) by both patients and students. This while the other three questionnaires scored higher, and thus as less easy/more difficult to complete. These scores are relative because the highest score still means a little more difficult but does not indicate `very difficult’. There was some difference in kurtosis. The distribution of the answers shows that the SERATS is more left-divided, while the other questionnaires show more distribution that leans to the right. This means that more people found the SERATS easier while this is the opposite with the other questionnaires.

**Table 4:** Frequencies of the experienced difficulty to complete.

	M/SD	SERATS	EES	ERS-ACA	HUMS
Patients (n = 179)	Mean	4.58	5.81	5.63	5.16
	Standard deviation	2.15	2.18	2.14	2.04
Students (n = 53)	Mean	4.09	5.52	5.37	5.42
	Standard deviation	2.12	2.10	2.16	2.03

## Discussion

The SERATS was developed as art therapy lacked outcome measures that could be used to monitor the specific effects of art therapy. Although the SERATS showed good psychometric properties in earlier studies, it lacked convergent validity and thus construct validity. The objective of this study was to test the convergent validity of the SERATS (its correspondence with measures of a similar construct). Therefore, three other questionnaires focused on emotion regulation and self-expression were selected, the EES (Emotional Expressivity Scale), the Emotion Regulation Strategies for Artistic Creative Activities Scale (ERS-ACA) and the Healthy-Unhealthy Music Scale (HUMS). In total 232 participants completed the four questionnaires: patients with emotion regulation problems (PDs) and a healthy student population. The SERATS showed again a high reliability in patients diagnosed with personality disorders (α = .90). The SERATS was positively associated with ERS-ACA *approach strategies* and *self-development strategies* in both patients and students. This means that what the SERATS measures is highly associated with emotion regulation strategies like acceptance, reappraisal, discharge and problem solving and with improving a sense of self including self-identity, increased self-esteem and improved agency.

The student sample showed a relatively high but negative correlation between SERATS and emotional expressivity (EES). This seems counter intuitive. However, looking at the items in the EES the central theme is the level of extravert emotional functioning. The EES is not linked to making art. A higher score means more emotional expressive in terms of general behavior. The student sample concerns art therapy students. They are generally more introvert and they prefer expression in the artistic medium rather than be verbally expressive. This could explain this negative correlation at this point.

There were some differences between the two groups: patients showed a higher correlation between the SERATS and the HUMS *healthy scale* while this was not the case in students. The student sample shows a high correlation between the SERATS and the ERS-ACA *avoidance strategies* while patients show a very low correlation at that point. Maybe this could be explained as a healthy strategy that students can manage but patients are struggling with. Given the scores, students seem to be able to avoid negative emotions when they want to. They seem to be able to avoid their fear, take another perspective or control their attention in a more positive direction while patients seem to have far more difficulties with this. They seem to fuse with their unwanted, negative emotions instead of defusing from them while making art. Avoidance seems to be an interesting goal to focus on for patients in therapy to gain a better emotion regulation. Given the scores, patients use avoidance (detachment, distraction and suppression) to a much lesser effective degree in their struggle with emotions. In turn, the patient sample shows a higher association between the SERATS and the HUMS *healthy scale* than the student sample. This could indicate that the positive, healthy side of art making is valued more by patients than by students and that they may need it more or use it for their self-expression and emotion regulation. The respondents rated the SERATS as relatively easy to complete compared to the other three questionnaires.

In conclusion, the SERATS is a useful and user-friendly tool for monitoring the effect of art therapy that is indicative of making art in a healthy way that serves positive emotion regulation and self-development.

A strength of the present study is the inclusion of specific chosen measures to test for convergent validity. The measures we used focus on emotion regulation and self-expression, and the context of art making, and were therefore fitting the objective of this study well. Another strength is that we developed and tested our scale in and with the effort of the target group of PD patients in specialized mental health care practice and also included a healthy population. The ERS-ACA focuses on artistic creative activities, `design or crafts’ activities including gardening, baking or cooking as ‘artistic’ activities, rather than art therapy and it involved a general healthy sample and not a clinical sample. Therefore, the SERATS seems to be better suited to patients with self-regulation and emotion regulation problems.

A limitation of the SERATS is that it is related to the specific context of art therapy. Taking this into account, the SERATS does not offer a zero measurement: the patient is questioned about art therapy and must experience this therapy at least during one or two sessions to have a first impression. Only when patients encountered the intervention earlier it can be used as a zero measurement. At the same time, a pure zero-measurement may be problematic more often because patients with personality disorders often already have a longer history of various treatments at start. Another limitation is that the SERATS does not cover all possible aspects of the art therapy situation but focuses only on self-expression and emotion regulation. Aspects like ability of structuring, the therapeutic relation, reality testing, or the experience of a number of different materials are not measured by the SERATS. However, the meaning of the SERATS comes forward is that it catches self-expression and emotion regulation. These are relevant and also transdiagnostic aspects of many mental health problems like impulse control and emotion regulation and anxiety. The results in this study seem to stress the SERATS as focusing on the healthy side of art making.

Emotion regulation implies the recognition and acceptance of emotions, problem solving and reappraisal, which appear to be protective against psychopathology (opposite of dysregulation: suppression, avoidance and rumination) [27, 55, 56]. In literature, emotion regulation is increasingly considered a central component of mental health given that it drives individuals’ abilities to manage their emotional experiences and adapt to daily life, and has been shown to influence a number of mental health conditions [57].

The use of the SERATS to monitor and measure art therapy can serve different goals. It supports further research on art therapy and its effects on self-expression and emotion regulation and enhance our understanding about how art therapy affects mental health. This could also shine some light on the added value of art therapy in multidisciplinary treatment programs. Due to the context-specific questions in the SERATS, it can evaluate art therapy progress in a multidisciplinary program, although one will have to be careful in establishing a causal relationship because this requires a stricter research design.

The implications and considerations for the use of the SERATS for art therapists working with people struggling with self-expression and emotion regulation problems, can be found on different levels. Art therapists can use it as a tool for reflection on their therapeutic efficacy and they can use outcomes to be discussed with their patients. The use of feedback in treatment can empower patients. It increases the sense of ownership of their own therapeutic change process, and it may result in faster progress or may be especially effective for `not on track’ patients [58]. This can stimulate the therapeutic relationship. PD patients often lack a sense of ownership due to self-related problems. For these patients, discussing results with their therapist may stimulate self-insight and `reframing’ their very often negative self-image [59]. It may improve treatment adherence and the therapeutic alliance [60, 61]. Poor treatment adherence is an often-described central challenge in PD patients. Social desirability in completing the questionnaire cannot be completely ruled out. Social desirability is a known problem of self-reporting questionnaires. However, there is no reason that this would be the case by how the SERATS was drafted. One could reason that feedback sessions with patient can be organized when some attention is paid to the possibility of social desirable answers. Such was however not performed in the current study.

Using the SERATS promotes participation of the patient and can lead to adjustment and acceleration of the therapy [62, 63]. Patients and art therapists involved in earlier studies mentioned that the SERATS meets the standards of clarity and readability, is specific for art therapy and usability in practice [24]. The preferred frequency for the use of the SERATS is three months; more frequent use would interfere with the therapy and less frequent seemed less useful. Discussing the results with patients was most helpful with patients who did not speak out easily or with who the therapeutic relationship was difficult. Art therapists mentioned that it stimulated awareness and reflection in patients which matched well the treatment goals. A possible limiting factor is the involved time investment.

Measuring outcomes of art therapy is important for several reasons and the SERATS offers objective, reliable and valid information. As an external assessment on art therapy it offers a therapeutic value in practice when making use of the feedback in the therapeutic relationship, it makes it possible to monitor effects of art therapy and contribute to quality improvement of art therapy. Doing so, the SERATS contributes to the improvement of mental health care aimed at a healthy emotional functioning for patients with severe self-expression and emotion regulation problems.

In short, we developed the SERATS as a brief, one-factor instrument that measures self-expression and emotion regulation in art therapy. The instrument consists of nine items scored on a 5-point Likert scale running from never true (1) to almost always true (5). The reliability is excellent (α = .94) and test-retest reliability is high (r = .96), despite it being a short instrument. The SERATS is sensitive to change in art therapy. The SERATS shows construct validity; discriminant validity and also convergent validity in relation to other questionnaires about self-expression and emotion regulation in the context of art making. The SERATS is a useful and user-friendly tool for monitoring the effect of art therapy that is indicative of making art in a healthy way that serves positive emotion regulation and self-development.

## Supporting information

S1 Appendix (DOCX)
