# Supplementary material for: Validity of the Self-Expression and Emotion Regulation in Art Therapy Scale (SERATS)
Source: PLoS One. 2021 Mar 10;16(3):e0248315. doi: 10.1371/journal.pone.0248315 (PMC7946186; doi:10.1371/journal.pone.0248315)
Supplement: S1 Appendix — (DOCX) [file pone.0248315.s001.docx]

**S1 Appendix.**

**SERATS**

**Self-expression and Emotion Regulation in Art Therapy Scale**

*©Haeyen, Van Hooren, Van der Veld & Hutschemaekers 2017*

| Name:    *(your data will be processed anonymously)* |
| --- |
| Age :                                                               Sex: M / F* |
| Date: |
| Treatment setting: Clinic / Multi-day part-time program / Outpatient* |
| Treatment Form: Group / Individual / Both* |

** Delete if not applicable*

*INSTRUCTION*

*The following statements will to a greater or lesser extent statements apply to you. Please read the statements carefully and answer them right away by ticking the chosen answer. Do not skip questions.*

|  | **Item** | 1. Never true | 2. Seldom true | 3. Some-times true | 4. Often true | 5. (Almost) always true |
| --- | --- | --- | --- | --- | --- | --- |
| 1 | I get in touch with my feelings through the process of making art |  |  |  |  |  |
| 2 | I am able to depict my feelings in art therapy |  |  |  |  |  |
| 3 | Through the process of making art, I am able to discover what is at play within me |  |  |  |  |  |
| 4 | I am able to express my feelings through the process of making art |  |  |  |  |  |
| 5 | I am able to make things fall into place in the art |  |  |  |  |  |
| 6 | Making art is a kind of outlet for me |  |  |  |  |  |
| 7 | A piece of art I have created can help me hold on to a particular feeling |  |  |  |  |  |
| 8 | I apply the new behavior that I have been experimenting with in art therapy outside of the therapy setting |  |  |  |  |  |
| 9 | I gain greater insight into my psyche through art therapy |  |  |  |  |  |
